# Traditional Chinese medicine for corona virus disease 2019: A protocol for systematic review

**DOI:** 10.1097/MD.0000000000021774

**Published:** 2020-08-28

**Authors:** Ju Huang, Liu Wu, Xiaodan Ren, Xinhui Wu, Yong Chen, Guoping Ran, Anming Huang, Liangxin Huang, Dan Zhong

**Affiliations:** a College of Acupuncture and Tuina, Chengdu University of Traditional Chinese Medicine; b Hospital of Chengdu University of Traditional Chinese Medicine, Chengdu; c The Second Affiliated Hospital of Army Medical University, Chongqing, China.

**Keywords:** corona virus disease 2019, systematic review, traditional Chinese medicine

## Abstract

**Background:**

Assessing the effectiveness and safety of Traditional Chinese medicine for treating patients with corona virus disease 2019 (COVID-19) is the main purpose of this systematic review protocol.

**Methods:**

The following electronic databases will be searched from inception to April 2020: Cochrane Central Register of Controlled Trials, PubMed, Web of Science, EMBASE, China National Knowledge Infrastructure, Traditional Chinese Medicine, Chinese Biomedical Literature Database, Wan-Fang Database, and Chinese Scientific Journal Database. All published randomized controlled trials in English or Chinese related to Traditional Chinese medicine for COVID-19 will be included. Primary outcomes are time of disappearance of main symptoms and serum cytokine levels. Secondary outcomes is Accompanying symptoms disappear rate, negative COVID-19 results rate on 2 consecutive occasions CT image improvement, average hospitalization time, occurrence rate of common type to severe form, clinical cure rate, and mortality. Two reviewers will conduct the study selection, data extraction, and assessment independently. The assessment of risk of bias and data synthesis will be conducted with Review Manager Software V.5.2.

**Results:**

The results will provide a high-quality synthesis of current evidence for researchers in this subject area.

**Conclusion:**

The conclusion of our study will provide evidence to judge whether traditional Chinese medicine is an effective intervention for COVID-19 patients.

**PROSPERO registration number:**

CRD42020181006.

## 1 Introduction

Companied with several cases of severe pneumonia with unknown pathogen were reported from Wuhan (Hubei, China) in December 2019, Corona Virus Disease 2019 (COVID-19) has been beginning its spreading and transferring.^[1]^ The disease has been named as COVID-19 by World Health Organization (WHO), which has resulted in an unprecedented chaos in recent decades.^[2]^ Based on the evidence of a rapidly increasing incidence of infections^[3]^ and the possibility of transmission by asymptomatic carriers,^[4]^ the SARS-CoV-2 can be transmitted effectively among humans and exhibits high potential for a pandemic.^[5–7]^ Nowadays, the advanced and convenient global transportation is the best accomplice with the epidemic, making it spread more effectively and fast. On January 30, 2020, the WHO declared the Chinese outbreak of COVID-19 to be a Public Health Emergency of International Concern.^[2]^

So far, there has been no effective treatment of COVID-19. Several potential drug candidates, including lopinavir/ritonavir (Kaletra), nucleoside analogs, neuraminidase inhibitors, remdesivir, umifenovir (arbidol), DNA synthesis inhibitors (such as tenofovir disoproxil, and lamivudine), chloroquine, and Chinese traditional medicine (such as ShuFengJieDu or Lianhuaqingwen capsules), have been proposed.^[8,9]^

It is perhaps clear that quarantine alone may not be sufficient to prevent the spread of COVID-19, and the global impact of this viral infection is 1 of heightening concern.

Traditional Chinese medicine (TCM), which originated in China, is used to maintain health and treat diseases.^[10]^ It includes medicinal herbs, prescriptions, Traditional Chinese medicine, tai chi and other contents. Chinese herbs have been used to treat human diseases in China for thousands of years.^[11]^ The awarding of part of the 2015 Nobel Prize in Physiology or Medicine for Tu Youyou s discovery and development of artemisinin, a potent antimalarial derived from the herbal Artemisia annua, is a clear example and reminder of the potential held by herbal medicine.^[12]^

According to incomplete statistics, there have been more than 300 epidemics recorded in China's history.^[13]^ In the struggles against epidemics over thousands of years, many methods have been proved effective in practice.^[14]^ Traditional Chinese medicine plays an extremely important role in the processes. Studies have shown that Artemisiae Argyi Folium, Atractylodis Rhizoma and Radix Angelicae Dahuricae were used for prevention of plagues by Chinese ancients.^[14]^ Peking University Shenzhen Hospital utilized an air disinfection technique combined atractylodus fumigate with chemical and physical disinfectants, leading to a no nosocomial infection report in the fight against SARS in 2003.^[15]^ Existing studies applaud the application of Chinese herbal medicine fumigation in clinical practices, and there are pharmacological studies confirmed its active ingredients.^[16,17]^ Chinese herbal medicine provides a new way for the combat to current severe COVID-19.

In China, Chinese medicine (CM) is proposed as a treatment option by national and provincial guidelines with substantial utilization.^[18]^ During the outbreak of SARS, CM was introduced as a treatment option since the early situation of epidemic in China. There are 3 clinical trials have proved the effectiveness of CM in the treatment of SARS.^[19]^ The sixth version of China national clinical guideline on COVID-19 associated pneumonia was published on February 18, 2020, the national guideline divided COVID-19 into medical observation period and treatment period. The recommended formulation during medical observation period involved huo-xiang-zheng-qi capsule and jin-hua-qing-gan granules, lian-hua-qing-wen capsule or shu-feng-jie-du capsule. The treatment period is divided into 4

clinical stages with the corresponding recommended prescription.^[20]^ Over 85% of confirmed cases involved CM use nationally (Wuhan over 67%)^[21]^ and the first CM-oriented designated Module Hospital in Wuhan operated since 14 February 2020.^[22]^ The clinical evidence of CM on COVID-19 is far from conclusive, but may be a good additional candidate at least for trial treatment considering the limited options available for COVID-19.^[18]^ Early reported benefits of CM included symptomatic relief, shortening fever duration, reverting radiological changes, and shortening hospital stay.^[23,24]^

Although there are lots of encouraged outcomes of TCM in the fights against COVID-19 in China. It still needs a systematic assessment to provide a convincing results for the application of TCM in the treatment of COVID-19 due to the incomplete evidences of present studies.

## 2 Methods

### 2.1 Study registration

The systematic review protocol has been registered in PROSPERO. The registration number: CRD42020181006, the consent of this protocol report is based on the Preferred Reporting Items for Systematic Reviews and Meta-Analyses Protocols statement guidelines.^[25]^

### 2.2 Inclusion criteria for study selection

#### 2.2.1 Type of study

We will include articles related to Traditional Chinese medicine therapy of patients for COVID-19. Due to language restrictions, we will search for articles in English and Chinese in order to get a more objective and true evaluation, all articles included are randomized controlled trial (RCT) type articles.

#### 2.2.2 Type of participant

All patients for COVID-19 will be included regardless of sex, age, race, education, and economic status. Pregnant women, postoperative infections, psychopaths, patients with severe cardiovascular and liver and kidney diseases will not be included.

#### 2.2.3 Type of intervention

Traditional Chinese medicine therapy including Chinese herbal medicine and its prescription, while other traditional Chinese therapies such as tuina, moxibustion, cupping and acupuncture will be excluded. We will compare the following interventions: treatments other than Traditional Chinese medicine (eg, usual or standard care, placebo, wait-list controls).

#### 2.2.4 Type of outcome measure

Primary outcomes: Time of disappearance of main symptoms (including fever, asthenia, cough disappearance rate, and temperature recovery time), and serum cytokine levels. Secondary outcomes: Accompanying symptoms (such as myalgia, expectoration, stuffiness, runny nose, pharyngalgia, anhelation, chest distress, dyspnea, crackles, headache, nausea, vomiting, anorexia, diarrhea) disappear rate, negative COVID-19 results rate on 2 consecutive occasions (not on the same day), CT image improvement, average hospitalization time, occurrence rate of common type to severe form, clinical cure rate, and mortality.

### 2.3 Data sources

The following electronic databases will be searched from inception to April 2020: the Cochrane Central Register of Controlled Trials (CENTRAL), PubMed, EMBASE, Web of Science, China National Knowledge Infrastructure, Chinese Biomedical Literature Database, and Wan-Fang Database. About other sources, we also plan to manually search for the unpublished conference articles and the bibliography of established publications.

### 2.4 Search strategy

The search terms on PubMed are as follows: Traditional Chinese medicine (eg, “Chinese medicine” or “herbs” or “TCM”); COVID-19 (eg, “Corona Virus Disease 2019” or “Corona Virus”); randomized controlled trial (eg, “randomized” or “randomly” or “clinical trial”). Combinations of Medical Subject Headings (MeSH) and text words will be used. The same search term is used in electronic databases in China. These search terms are shown in Table 1.

**Table 1:**
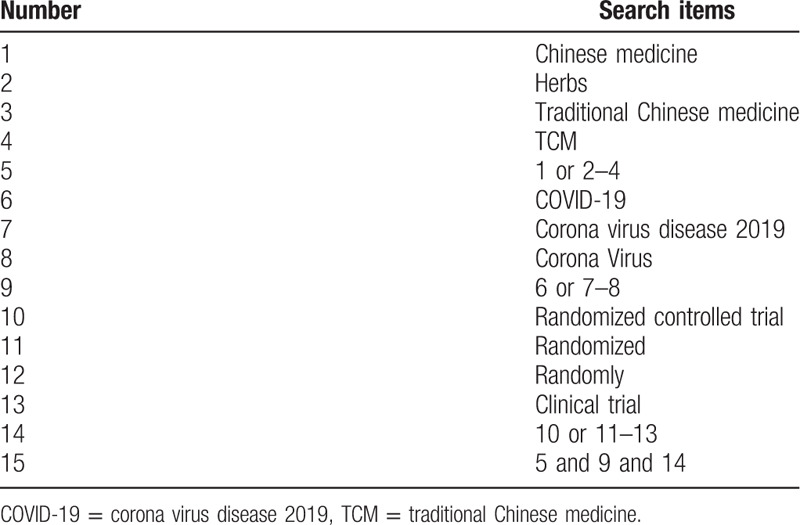
Search strategy for the PubMed database.

### 2.5 Data collection and analysis

#### 2.5.1 Selection of studies

**Figure 1:**
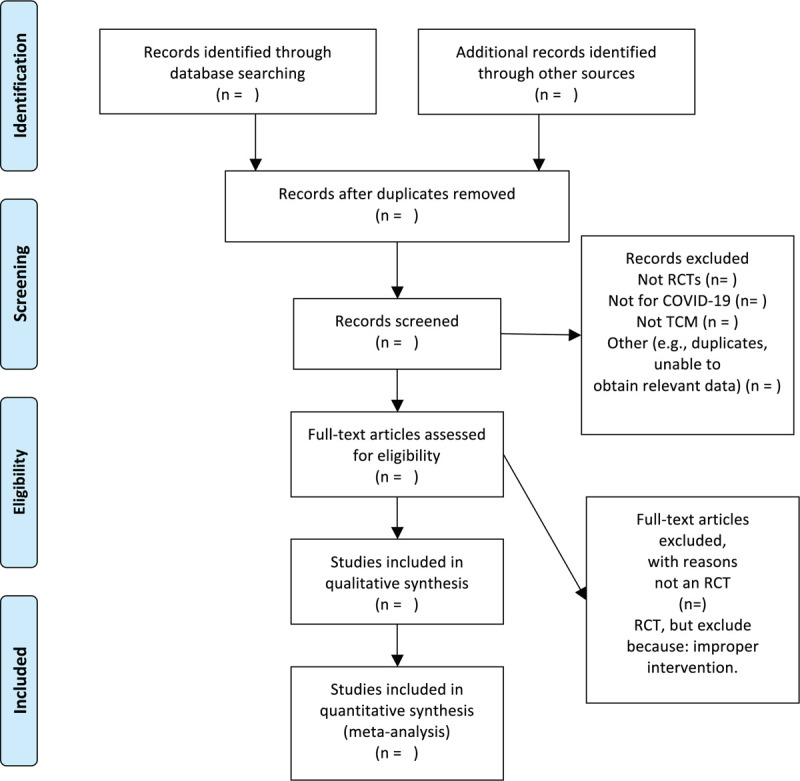
Flow chart of the study.

We chose the the preferred reporting items for systematic reviews and meta-analyses flow chart to show the process of selecting literature for the entire study (Fig. 1). Before searching the literature, all reviewers will discuss and determine the screening criteria. After the screening requirements are clearly defined, the 2 reviewers will independently review and screen the literature. They screened the titles and abstracts of the literature, in order to get qualified studies, and then excluded some duplicate studies or studies with incomplete information. We will also try to obtain the full text, and the obtained literature will be managed by using EndNote software, V.X8 (United States). In case of disagreement between the 2 reviewers, discussions will be held with the third author for arbitration.

#### 2.5.2 Data extraction and management

The authors will strictly follow the inclusion criteria and select RCT articles related to the topic. Through the analysis of the article, we know participants’ characteristics (height, weight, sex), interventions, outcomes, the study characteristics (press, nationality, journals, research design), adverse reactions, etc. If there is any disagreement between the 2 authors in the literature data extraction, a third article participant will discuss the decision together. If there is a lack of data in the literature, we will contact the author or publisher as much as possible.

#### 2.5.3 Assessment of risk of bias in included studies

We will use the Cochrane collaborative tool to independently assess the risk of bias in the included studies. We will evaluate the following aspects of the article: sequence generation, assignment sequence hiding, blindness of participants and staff, outcome evaluators, incomplete result data, selective result reporting, and other sources of bias. The risk of bias is evaluated at 3 levels, namely, low risk, high risk, and ambiguity. If the information is vague, we will try to contact the author of the article.

#### 2.5.4 Measures of treatment effect

In this protocol, we will use 95% confidence interval risk ratio (RR) to rigorously analyze the dichotomous data. And for the continuous data, mean difference (MD) or standard MD (SMD) is used to measure the efficacy of 95% confidence interval.

#### 2.5.5 Unit of analysis issues

We will include data from parallel group design studies for meta-analysis. In these trials, we will collect and analyze individual measurements of each outcome for each participant.

#### 2.5.6 Management of missing data

We will try our best to ensure the integrity of the data. If the included RCT data is not complete, we will try every means to contact the corresponding author of the article, including sending emails or making a phone call. If the corresponding author cannot be contacted, we will remove the experiment with incomplete data. After data integrity is assured, intention analysis therapy and sensitivity analysis will be performed.

#### 2.5.7 Assessment of heterogeneity

For the detection of heterogeneity, the *I*^2^ test will be used to detect the heterogeneity among trials. When the *I*^2^ test value is < 50% and *P* value > 1, we think there is no heterogeneity between these trials, and when the *I*^2^ test value is > 50% and the *P* value is < 1, there is significant heterogeneity between these included trials. If significant differences are detected, we will analyze the possible causes of heterogeneity, and then we can use the random effects model.

#### 2.5.8 Assessment of reporting biases

In this analysis, once >10 trials are included, funnel plots could be used to test for reporting bias.

#### 2.5.9 Data synthesis

We will use Review Manager Software V.5.3 (Copenhagen, Denmark) for data analysis and quantitative data synthesis. If there is no finding of statistical heterogeneity, the fixed-effect model is used for data synthesis. If there is significant statistical heterogeneity, we will use the random effect model, and all participants will explore the possible causes from a clinical and methodological perspective and provide a descriptive or subgroup analysis.

#### 2.5.10 Subgroup analysis

Subgroup analysis will be performed to explain heterogeneity if possible. Factors such as different types of control interventions and different outcomes will be considered.

#### 2.5.11 Sensitivity analysis

Based on sample size, study design, heterogeneous quality, methodological quality, and statistical model, sensitivity analysis will be performed to exclude trials with quality defects and ensure the stability of the analysis results.

#### 2.5.12 Grading the quality of evidence

This paper will use the evidence quality rating method to evaluate the results obtained from this analysis. GRADE is generally applied to a large amount of evidence. It has 4 evaluation levels, namely, high, medium, low, and very low. GRADE was used to evaluate the bias, inconsistencies, discontinuities, and inaccuracies of test results. In the context of the system review, quality reflects our confidence in the effectiveness of assessment.^[26]^

#### 2.5.13 Ethical review and informed consent of patients

Ethics and dissemination: The content of this article does not involve moral approval or ethical review and will be presented in print or at relevant conferences.

## 3 Discussion

This review is divided into 4 parts: identification, literature inclusion, data extraction, and data synthesis. It will systematically review the RCT literature, this review will evaluate the effectiveness of Traditional Chinese medicine in treating COVID-19 convalescent patients. There are also limitations in our research and the language bias here is that we only search for Chinese and English documents. Our study may provide a basis for clinicians to choose replacement therapy for further study in the future.

## Author contributions

All authors have read and approved the final manuscript.

**Data curation**: Ju Huang, Liu Wu, Xiaodan Ren.

**Funding acquisition**: Dan Zhong.

**Investigation**: Xinhui Wu.

**Methodology**: Yong Chen. Software: Guoping Ran, Anming Huang, Liangxin Huang.

**Supervision**: Dan Zhong.

**Visualization**: Anming Huang.

**Writing – original draft**: Ju Huang, Liu Wu, Xiaodan Ren.

**Writing – review & editing**: Ju Huang, Liu Wu, Xiaodan Ren, Dan Zhong.
